# Development of a new sternal dehiscence prediction scale for decision making in sternal closure techniques after cardiac surgery

**DOI:** 10.1186/s13019-021-01555-2

**Published:** 2021-06-14

**Authors:** Ehab Nooh, Colin Griesbach, Johannes Rösch, Michael Weyand, Frank Harig

**Affiliations:** 1 Department of Cardiac Surgery, University Hospital Erlangen, Friedrich- Alexander University Erlangen- Nuremberg, Krankenhausstr. 12, D-91054 Erlangen, Germany; 2 Institute of Medical Informatics, Biometry and Epidemiology (IMBE), Waldstr. 6, D-91054 Erlangen, Germany

**Keywords:** Cardiac surgery, Sternal dehiscence, Prediction, Scoring system, Tailored technique

## Abstract

**Background:**

After sternotomy, the spectrum for sternal osteosynthesis comprises standard wiring and more complex techniques, like titanium plating. The aim of this study is to develop a predictive risk score that evaluates the risk of sternum instability individually. The surgeon may then choose an appropriate sternal osteosynthesis technique that is risk- adjusted as well as cost-effective.

**Methods:**

Data from 7.173 patients operated via sternotomy for all cardiovascular indications from 2008 until 2017 were retrospectively analyzed. Sternal dehiscence occurred in 2.5% of patients (*n* = 176). A multivariable analysis model examined pre- and intraoperative factors. A multivariable logistic regression model and a backward elimination based on the Akaike Information Criterion (AIC) a logistic model were selected.

**Results:**

The model showed good sensitivity and specificity (area under the receiver-operating characteristic curve, AUC: 0.76) and several predictors of sternal instability could be evaluated. Multivariable logistic regression showed the highest Odds Ratios (OR) for reexploration (OR 6.6, confidence interval, CI [4.5–9.5], *p* < 0.001), obesity (body mass index, BMI > 35 kg/m^2^) (OR 4.23, [CI 2.4–7.3], *p* < 0.001), insulin-dependent diabetes mellitus (IDDM) (OR 2.2, CI [1.5–3.2], *p* = 0.01), smoking (OR 2.03, [CI 1.3–3.08], *p* = 0.001). After weighting the probability of sternum dehiscence with each factor, a risk score model was proposed scaling from − 1 to 5 points. This resulted in a risk score ranging up to 18 points, with an estimated risk for sternum complication up to 74%.

**Conclusions:**

A weighted scoring system based on individual risk factors was specifically created to predict sternal dehiscence. High-scoring patients should receive additive closure techniques.

## Background

After the first description of the median sternotomy as an operative access to the mediastinum by MILTON in 1897, this is still the most common approach in cardiovascular surgery [1]. The evolution in modern cardiac surgery creates less and minimal invasive surgical approaches to the heart, but this approach is relatively safe and offers the best exposure in most of the cardiac surgical procedures [2]. As every surgical procedure bears the potential of complications, this approach also bears a potential risk of sternal dehiscence and infection that may threaten the outcome of the operation and potentially affect not only the patient’s physical but also his psychological recovery. It is also a matter of economics, as complicated postoperative courses are up to 2.8 times more expensive than uncomplicated cases [3, 4].

The incidence of surgical site infections was targeted in many studies trying to evaluate the risk factors [3–10] and an incidence of 3.5% for major infections was found [3]. Even risk score models were created [6–9], but they were mostly designed not for all indications but only for coronary patients and especially associated with the usage of bilateral internal thoracic arteries (BITA) in coronary artery bypass grafting (CABG) [10].

The complication of sternal instability alone is focused only in some studies and reported to occur in 3% (0.5–8.0%) of patients after sternotomy [11, 12]. In order to prevent those complications, pre-emptive risk-reducing surgical techniques like sternal rigid plate fixation have been evolved [13], and superior effects over simple wiring technique concerning bone healing, postoperative pain and overall costs could be shown in a multicentre randomized controlled trial (RCT) [4].

However, a general use of more expensive techniques is not always technically required nor economically justified. The standard sternal wires technique, which is applicable in the vast majority of cases, is fast and cost-effective. Nowadays, the increased morbidity of cardiac patients made the use of wires not as effective as before. Alternative techniques to overcome this surgical challenge comprise special wire techniques (e.g. Robicsek’s figure of eight, [11]), the usage of metallic bands (Parham bands [14]) and finally the technique of rigid plates fixation using titanium plates and screws [4]. These closure techniques are definitely more effective but on the other hand time-consuming and expensive [13]. The identification of patients who are at high risk for sternal instability and consequently adopting the suitable surgical technique is the real surgical challenge. Therefore, it is necessary to develop a risk model to predict the risk of sternal dehiscence.

In the present study, the authors have reviewed retrospectively their surgical experience over the last 10 years with sternal complications after all-type cardiothoracic operations. The aim of the study was to develop a risk model to predict sternal dehiscence in surgical patients who may receive special intervention strategies to reduce rates of sternal instability.

## Methods

### Study population

From 1 January 2008 throughout 2017, 8.615 adult consecutive patients with all-type heart disease underwent cardiac surgery through median sternotomy at the authors’ institution, a quaternary, acute care university hospital in northern Bavaria. Most of the patients (61%) underwent CABG, 29% underwent valve surgery and 10% aortic surgery. Preoperative antibiotic prophylaxis was part of every patient’s standardized medical treatment.

After checking for inclusion (age over 18 years, surgery through median sternotomy) and exclusion criteria (lateral thoracotomy, complex sternal closure techniques), the data of a cohort of 7.173 patients were analysed (Table 1).

**Table 1:** Preoperative patients’ characteristics^a^

Characteristics	n	%	Range
	7173		
**Age (years)**			
Mean	66.5	(+ 12.4)	(22–85)
< 60	1878	26.2	
> 60	5295	73.8	
**Gender**			
Male	5306	74.0	
Female	1867	26.0	
**BMI (kg/m**^**2**^**)**			
Mean	28,1	(+ 4.6)	(14–57)
< 25	1861	25.9	
25–35	4791	66.8	
> 35	521	7.3	
**Diabetes mellitus**			
No	4978	69.4	
NIDDM	1356	18.9	
IDDM	839	11.7	
**COPD**^**b**^			
Yes	624	8.7	
No	6549	91.3	
**Renal impairment**^**c**^	190	2.6	
**Smoker**			
No	3854	53.7	
Former	2034	28.4	
Current^d^	1285	17.9	
**Previous cardiac OP**			
no	6096	85.0	
> 6 months	1077	15.0	
**Cardiogenic shock**			
No	6891	96.1	
< 3 weeks	82	1.1	
< 48 h	200	2.8	
**Surgical priority**			
Elective	3197	44.6	
Urgent	3111	43.4	
Emergency	865	12.1	
**OP-Duration (min)**			
Mean (SD)	214	(+ 83.5)	(17–1135)
< 300	6436	89.7	
> 300	737	10.3	
**Re-Exploration**			
Yes	420	5.9	
No	6753	94.1	

Legend: *BMI* Body mass index, *COPD* Chronic obstructive pulmonary disease

^a^Values are no. of patients or mean + SD with interquartile range in brackets

^b,c^all stages, Definition: see EuroSCORE II; ^d^within last 2 months preoperativel

### Human participant protection

This retrospective study complied with the Helsinki Declaration (2000) and approval to perform this analysis was given by the local Ethics Committee (EC, No.233_20 BC), based on retrospective data retrieval. For this reason, the EC waived the need for written patients’ informed consent.

### Data collection and follow-up

Within the cohort of 7173 patients, a subset of 176 (2.5%) developed a sternal dehiscence, regardless of a primary ‘aseptic’ dehiscence or secondary to an infection (septic dehiscence). We have deliberately omitted the post-operative risk factors, as they could not alter the decision of the sternal closure technique.

### Definitions and outcome measures

The definition of sternal dehiscence is a clinically noticeable mobility of the sternal parts that causes pain or abnormal respiratory movement and leads to a surgical intervention.

### Statistical analysis

For the variables listed, a multivariable logistic regression model was estimated and within this model, the variable “complication Yes vs. No”, was the dichotomous outcome. Subsequently, a backward elimination based on the Akaike Information Criterion (AIC) was used to select a logistic model best suitable for prediction. Odds ratios were calculated for each variable and the area under the curve (AUC) was determined with corresponding 95% confidence interval. The analysis was performed with the software R version 3.6.3 [15] by colleagues of the IMBE, Institute of Medical informatics, Biometry and Epidemiology of the Friedrich-Alexander-University Erlangen-Nürnberg (C.G., co-author and E.W., see acknowledgement). For the selection of the best model, the functions glm() and step() of the “stats” package were used, whereas the function roc() of the package “pROC” was used to compute the AUC.

Exclusion criterion was the primary use of any other complex techniques for sternal osteosynthesis.

## Results

### Risk factors for sternal dehiscence (malunion of the sternum) and uni-variable analysis

The data of 7173 patients were analysed and 176 (2.5%) patients were found to suffer from sternal dehiscence due to malunion of the sternum (*MUST*). These patients were compared to 6997 (97.5%) patients who did not develop a sternal complication.

The following factors were risk factors for *MUST* according to the univariable analysis (Table 2)

**Table 2:** Risk factors (univariable analysis)^a^ (*n* = 7173)

Variable	Sternal dehiscence		No sternal complication		***P***-value
	n	%	n	%	
	**176**	2.5	**6997**	97.5	
**Age (years)**					
Mean	66.9	(22–85)	66.5	(18–92)	
< 60	34	19	1819	26	
> 60	142	81	5178	74	0.0065
**Gender**					
Male	142	81	5178	74	0.0247
Female	34	19	1819	26	
**BMI (kg/m**^**2**^**)**					
Mean	30.3	(17–46)	28.1	(14–57)	
< 25	27	15.4	1816	26.0	
25–35	115	65.3	4691	67.0	
> 35	34	19.3	490	7.0	< 0.0001
**Diabetes mellitus**					
No	94	54	4884	69.8	
NIDDM	40	22	1316	18.8	0.1408^b^
IDDM	42	24	797	11.4	< 0.0001^c^
**COPD**^**d**^					
Yes	29	16.5	595	8.5	0.0507
No	147	83.5	6402	91.5	
**Renal failure**^**e**^					
Yes	7	4	183	2.6	0.0543
No	169	96	6814	97.4	
**Smoker**					
No	73	42	3779	54.0	
Ex-	61	35	1976	28.2	0.0546
Current	42	23	1242	17.7	0.0009
**Previous cardiac OP**					
No	154	87.5	5943	84.9	
> 6 months	22	12.5	1054	15.1	0.0533
**Cardiogenic shock**					
No	171	97.2	6730	96.2	
< 3 weeks	2	1.1	80	1.1	0.7441^f^
< 48 h.	3	1.7	187	2.7	0.1124^g^
**Surgical priority**					
Elective	69	39	3128	44.7	
Urgent	79	45	3032	43.3	0.6494^h^
Emergency	28	16	837	12.0	0.1169^i^
**OP-Duration**					
Mean (SD)	236	+ 130			
< 300 min	148	84	6288	89.9	
> 300 min	28	16	709	10.1	0.1626
**Re-Exploration**					
Yes	47	27	373	5.3	< 0.0001
No	129	73	6624	94.7	

Legend: The incidence of each variable is shown in the group with sternal dehiscence and with no sternal complication. Univariable analysis was performed and *P*-value was calculated

^a^Values are no. of patients or mean + SD with interquartile range in brackets; *BMI* Body Mass Index, *NIDDM* Non Insulin Dependent Diabetes mellitus, *IDDM* Insulin dependent Diabetes mellitus; ^b^NIDDM vs. no DM; ^c^IDDM vs. no DM; COPD: Chronic obstructive pulmonary disease; ^d,e^all stages, as defined by EuroScore II; ^f^Cardiogenic shock within 3 weeks vs. no; ^g^Cardiogenic shock > 48 h vs. no; ^h^elective vs. urgent; ^I^elective vs. emergency

Older age (age > 60 y), male gender, severe obesity (BMI ≥35 kg/m^2^), insulin-dependent diabetes mellitus (IDDM), current smoking (inhalative tobacco use within 2 months preoperatively), previous cardiac operation and rethoracotomy for re-exploration of the mediastinum.

### Risk factors for sternal dehiscence (malunion of the sternum) and multi-variable analysis

Using these dependent risk factors for MUST, a multi-variable analysis model was developed to examine preoperative risk factors. The following were risk factors for MUST according to the multi-variable analysis (Table 3)

**Table 3:** Risk factors for sternal instability (multivariate analysis) (*n* = 7173)

Factor	Preoperative evaluation		
	OR	(95% CI)	*P*-value
Re-exploration	6.61	(4.51–9.54)	< 0.001
BMI ≥35 kg/m^2^	4.23	(2.46–7.34)	< 0.001
IDDM	2.24	(1.50–3.29)	0.010
Smoker (within 2 months preop.)	2.03	(1.32–3.08)	0.001
Age > 60 y	1.77	(1.19–2.72)	0.007
BMI ≥25–35 kg/m^2^	1.66	(1.09–2.62)	0.022
Male gender	1.56	(1.00–2.37)	0.030
Previous cardiac OP	0.60	(0.36–0.95)	0.037
Smoker (former)	1.44	(1.44–2.08)	0.051
COPD	1.53	(0.97–2.33)	0.059
Cardiogenic shock (< 48 h preop.)	0.38	(0.09–1.07)	0.112
Priority: emergency	1.48	(0.89–2.39)	0.117
NIDDM	1.36	(0.91–1.99)	0.126
OP Duration > 5 h	1.38	(0.86–2.15)	0.163
Renal impairment (all stages)	1.42	(0.58–2.98)	0.398
Priority: urgent	1.08	(0.77–1.51)	0.649
Cardiogenic shock (< 3 weeks preop.)	0.79	(0.13–2.63)	0.740

*OR* Odds ratio, *CI* Confidence intervall, *BMI* Body mass index, *IDDM* Insulin-dependent diabetes mellitus, *COPED* Chronic obstructive pulmonary disease, *NIDDM* Non-insulin-dependent diabetes mellitus

Re-exploration of the mediastinum (e.g. for bleeding), severe obesity (BMI ≥35 kg/m^2^), IDDM, current smoking (inhalative tobacco use within 2 months preoperatively), older age (age > 60 y), moderate obesity (BMI ≥25 and < 35 kg/m^2^), male gender, previous cardiac operation and former smoking (< 2 months before OP), COPD.

### The new predictive scoring system for malunion of the sternum after cardiac surgery

We created a model of a new scoring system (MUST-Score) to predict mal-union of the sternum after sternotomy for cardiac surgery according to the multivariable analysis model. After performing backward selection on the full model (Table 3), the remaining factors coefficient estimates were scaled to the largest one being 5 and rounded to the nearest whole (Tables 4 and 5). This resulted in a risk score between − 1 and 18, reflecting an estimated risk for sternum complication of 0.22 to 73.65%, while the average risk is 2.5%.

**Table 4:** The scoring of the risk factors for MUST

Risk factor	Points
Re-exploration	5
BMI > 35 kg/m^2^	4
Age > 60 years	2
IDDM	2
Smoker: current	2
Male gender	1
COPD	1
OP Duration > 300 min	1
BMI 25–35 kg/m^2^	1
NIDDM	1
Smoker: former	1
Previous cardiac OP > 6 months	−1

*MUST* Mal-Union of the Sternum, *BMI* Body Mass Index, *IDDM* Insulin dependent diabetes mellitus, *COPD* Chronic obstructive, Pulmonary disease, *NIDDM* Non-insulin dependent diabetes mellitus

**Table 5:** The total score and expected risk of MUST

Total score	Expected risk of MUST (%)
-1	0.2
0	0.3
1	0.5
2	0.7
3	1.0
4	1.4
5	2.1
6	3.0
7	4.3
8	6.1
9	8.7
10	12.2
11	16.8
12	22.7
13	29.9
14	38.3
15	47.5
16	56.9
17	65.8
18	73.7

*MUST* Mal-Union of the STernum

### The accuracy of prediction

**Fig. 1:**
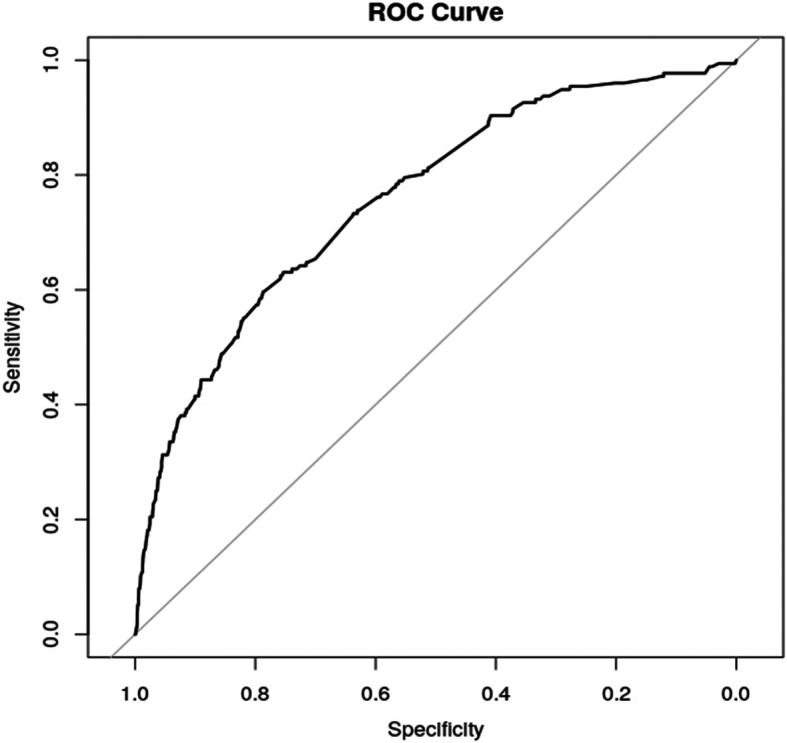
The predictive scoring system for MUST after sternotomy for cardiac surgery. Legend: The accuracy of prediction of the model was assessed with the receiver-operating characteristic curve (ROC) and the calculation of the area under the receiver-operating characteristic curve (AUC). The AUC was calculated 0.76 [95% CI: 0.72; 0.80]. AUC: Area under the curve. ROC Curve: Receiver Operating Characteristic curve. MUST: Mal-Union of the Sternum

The accuracy of prediction was assessed with the receiver-operating characteristic curve (ROC) and the calculation of the area under the receiver-operating characteristic curve (AUC) (Fig. 1).

In this model, the AUC was calculated 0.76 [95% CI: 0.72; 0.80]. The scoring system based on this model showed an AUC of 0.75 [95% CI: 0.72; 0.79].

According to arbitrary definitions of *Sweats* [16], the accuracy of prediction can be defined as moderate (AUC: 0.7–0.9).

## Discussion

Sternal dehiscence after sternotomy for cardiac operations severely affect the patients’ physical and psychological recovery. It is also a matter of economics, as complicated postoperative courses are up to 2.8 times more costly than uncomplicated cases. The complication of sternal instability alone is discussed only in some studies and reported to occur in 3% (0.5–8.0%) of patients after sternotomy [11, 12]. Preventive strategies comprise the usage of vacuum-assisted wound closure therapy, special surgical techniques to reinforce the osteosynthesis and the development of models to identify patients at high risk for sternal wound complications.

Several models focus on special surgical settings like the usage of bilateral internal thoracic artery grafting in coronary artery bypass grafting [10]. Moreover, some models were created to prevent infections on multiple surgical sites (sternum, legs) [3, 5], the development of infections alone [3, 5, 6] or in combination with sternal instability [13]. Although sternal wound complications have a complex pathogenesis depending on specific comorbidities as well as pre-, intra– and postoperative factors, we consider the sternal instability as the initial step towards further sternal wound healing complications including deep sternal wound infections and mediastinitis. In this context, the development of a model to predict the risk of sternal dehiscence may be useful to calculate patients’ risk rationally and individually and to target these patients in the operating room for special surgical intervention strategies.

The aim of this study was both to analyse the risk factors for sternal instability and develop a predictive risk score based on the results of this analysis. Surgeons who operate their patients via sternotomy would be able to evaluate the risk of sternum instability individually, and may optimize the sternal osteosynthesis technique. Up to now, there is no scoring system specifically created to predict the individual risk for sternal instability due to mal-union of the sternum (MUST).

Among all patients (*n* = 8.615) who underwent all-type cardiothoracic operations in a quaternary university hospital setting over a 10-years period from 2008 to 2017 mal-union of the sternum (MUST) occurred in 2.5% of the patients (*n* = 176).

Other authors reported a higher sternal complication rate for wire cerclage usage compared to rigid plate fixation of the sternum (5% vs. 0% after 6 months) [4]. Although rigid plate fixation was associated with a trend toward greater index hospitalization costs, 6-month follow-up costs tended to be lower. As a result, total costs were similar between groups.

*Ramann* et al. [13] investigated the sternal bone healing in a multicentre RCT and compared pain scores and narcotic usage in patients who received rigid plate fixation versus conventional wire cerclage. He could find a sternal union rate of 70% at 6 months in patients with rigid plate fixation compared to 24% in patients with sternal wiring. Pain scores and narcotic usages were significantly lower in the plate fixation group.

### Predictive scoring systems or computed scales for the occurrence of surgical complications

Those predictive scales are instruments to optimize patients’ outcome. Patients at a high risk for a complicated course may need more attention than patients at standard risk.

In 1992, *O’Connor* et al. [7] developed a multivariate clinical prediction rule using logistic regression analysis, a statistical method that allows the calculation of the conditional probability of death (in-hospital mortality associated with CABG surgery). The area under the ROC curve obtained from the training set of data was 0.74 (perfect, 1.0). The prediction rule performed well when used on a test set of data (area, 0.76). The correlation between observed and expected numbers of death was 0.99.

### Prediction of major infections after cardiac surgery

Other studies focused on major all- site infections after cardiac surgery. As published in 2005, *Fowler* et al. [3] analysed 331.429 patients (operated 2002–2003 for CABG) from the STS National Cardiac Database to identify risk factors for major infection. A simplified risk scoring system of twelve variables accurately predicted risk for major infection (overall, 3.5%; of them, 25% mediastinitis, 33% saphenous harvest site infection, 35% septicaemia, 7% multiple sites). Patients with a major infection had a significantly higher mortality than patients without an infection (17.3% versus 3.0%).

*Hussey* et al. [5] performed a more specific analysis of sternal wound infections after cardiac surgery in 1998. In this study, a sternal wound infection prediction scale (SWIPS) was developed and further revised (SWIPS-R). A multivariate logistic regression with 12 risk factors provided up to 76% correct predictions.

The scoring systems were mainly considered for sternal wound infections generally after any cardiac operation [3, 5] or more specially like CABG surgery with one or bilateral use of the BITA, analysed by *Gatti* et al. [10]. With this special scoring system inaugurated by *Gatti*, deep sternal wound infection (DSWI) after BITA grafting, can be predicted with a accuracy of 0.72 to 0.73 (AUC of the ROC-curve).

Until now, the most effective prevention methods including scoring systems have not been found. They remain objects of an ongoing discussion.

In a review of 2002, *Losanoff, Richmann and Jones* [17], described not only preoperatively known risk factors, such as insulin-dependent diabetes mellitus, chronic obstructive pulmonary disease, body- mass- index above 35 kg/m^2^ (BMI > 35 kg/m^2^), obesity in diabetic women. He also described intraoperative factors like prolonged bypass time, sternal devascularisation (by internal thoracic artery use) and harvesting technique (whether skeletonized or pedicled technique). Additionally, the authors pointed out that suboptimal primary sternal closure should be considered a significant intraoperative risk factor. This explains the necessity of a predictive scoring system that may help the surgeon in decision –making concerning the most appropriate sternal closure technique. This is called ‘a tailored approach’ in a publication of *Nenna* et al. [12]. They proposed a decisional algorithm based on clinical experience. The definition of high-risk patients comprises COPD, obesity, BITA use, diabetes, off-midline sternotomy. An algorithm for tailored sternal closure techniques is based on patients’ risk factors and the surgeons’ experience. The authors of this review are aware of the absence of consensus in the literature and the severe lack of RCT regarding the optimal sternal closure method.

Our scoring model is based on a multivariable logistic regression model, it predicts a sternal dehiscence more precisely than other scoring systems (AUC: 0.76). The risk factors are individually weighted and therefore, a more specific surgical technique can rationally be chosen and eventually higher costs are well justified.

### How to interpret the predictive scoring system (MUST-score)

In the first step, the patients diagnoses are weighted with points in a scale from − 1 to 5 (Table 4). Secondly, the total score represents the expected risk of sternal dehiscence within a range from 0.2 to 73.7% (Table 5).

The scaling is arbitrarily divided into a coloured traffic light scheme starting from

- green (score up to 4 points, representing an expected low risk of MUST below 1%), to
- yellow (score up to 8 points, intermediate risk of MUST below 5%), to
- red (score up to 11 points, high risk of MUST above 15%) (Table 6).

**Table 6:** Classification of risk groups and surgical technique modification

Score	Risk		Surgical technique
0	0.3%	low	**Standard**:
1	0.5%		Single wires
2	0.7%		
3	1.0%	< 1.0%	
4	1.4%	Intermediate	**Standard plus**:
5	2.1%		Special wiring techniques,
6	3.0%		e.g. figure of eight,
7	4.3%	< 5%	intercostal, double wires
8	6.1%	high	**Reinforced**:
9	8.7%		Wires plus bands or
10	12.2%	< 15%	rigid plates fixation^a^
11	16.8%	Very high	**Specially reinforced:**
12	22.7%		Rigid plates fixation (360-plates +bands^b^)
13	29.9%	< 30%	(minimum 3 plates, 5 bridges)
14	38.3%	Extremely high	Combination of plates and bands (360°)
15	47.5%	< 50%	other techniques:
16	56.9%	> 50%	Additional to Plates
17	65.8%		Retention sutures
> 18	73.7%		Vacuum-assisted wound closure

^a^SternaLock™ Blue, ^b^SternaLock™ 360

**Fig. 2:**
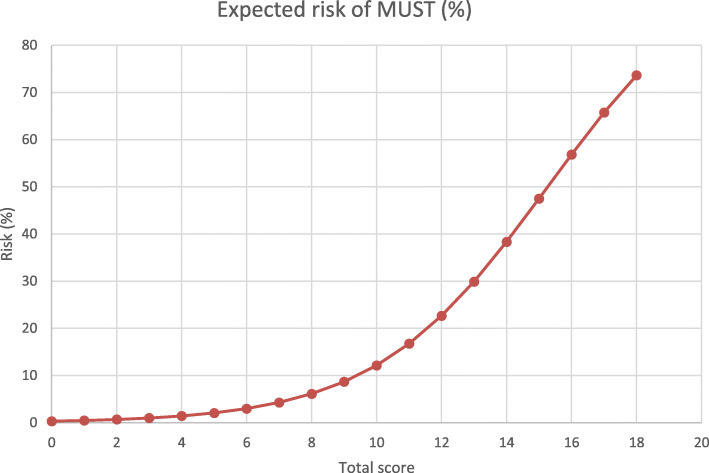
The relationship between the total score and the expected risk of MUST. Legend: The expected risk of Malunion of the sternum (MUST) is shown. The graph shows a nearly exponential curve on the range of 0–12 points, afterwards, the graph follows nearly a straight line

The expected risk of malunion increases with the total score. As shown in Fig. 2, the graph follows an exponential curve with an increase behind a score of 10. We consider the risk as high and recommend specially reinforced techniques for the sternal closure as described in Table 6.

The surgeon may preoperatively calculate the patients risk group and plan the modification of the sternal closure technique. We consider this a well-tailored approach with an underlying rational score that fits the patients’ needs as well as economical requirements.

### Study limitations

Even the best scoring system does not relieve the surgeon from correct judgement and proper technical quality of the employed technique. Several clinical circumstances may limit the guidance of scores and the usage of extended techniques, e.g. diffuse bleeding with a certain probability of postoperative reexploration and hemodynamic instability.

From the statistician’s point of view, the probabilities have to be interpreted with caution. The analysed complication (sternal dehiscence) is a rare event (2.5%), thus the logistic regression model is lacking of some accuracy in the marginal zones (nearby 0 and 100%). Exclusion criterion was the primary use of any other complex techniques for sternal osteosynthesis, so that some of the so-called high-risk patients potentially did not contribute to the complication group. As well as data of juvenile patients below an age of 18 years were not enrolled.

Experience and availability of special techniques cannot be taken for granted in each centre.

Further studies will have to validate the precision of the performance of this scoring system. In this future validation study, all data would be analysed that have been entered in the database after inauguration of the MUST-score in this department.

## Conclusion

A weighted scoring system based on individual risk factors was specifically created to predict sternal dehiscence. This risk score accurately identifies high-risk patients who may benefit form tailored interventions aimed at the reduction of this severe complication of cardiac surgery.

High-scoring patients should receive more than standard closure techniques. Rigid plates fixation techniques even in combination with bands and external supportive techniques like retention sutures, support vests or vacuum-assisted wound closure may complete the surgeon’s technical armament.

## Data Availability

Please contact corresponding author for data request.

## Abbreviations

AUC: Area under the curve
ROC Curve: Receiver Operating Characteristic curve
BITA: Bilateral internal thoracic arteries
CABG: Coronary artery bypass grafting
AIC: Akaike Information Criterion
MUST: Malunion of the sternum
BMI: Body Mass Index
NIDDM: Non- Insulin Dependent Diabetes mellitus
IDDM: Insulin dependent Diabetes mellitus
COPD: Chronic obstructive pulmonary disease
